# Development of a Predictive Model for Evaluation of the Influence of Various Parameters on the Performance of an Oscillating Water Column Device

**DOI:** 10.3390/s24113582

**Published:** 2024-06-01

**Authors:** Felice Sfravara, Emmanuele Barberi, Giacomo Bongiovanni, Massimiliano Chillemi, Sebastian Brusca

**Affiliations:** Department of Engineering, University of Messina, 98166 Messina, Italy; felice.sfravara@unime.it (F.S.); giacomo.bongiovanni@studenti.unime.it (G.B.); sebastian.brusca@unime.it (S.B.)

**Keywords:** OWC, machine learning, Savonius, predictive model

## Abstract

Oscillating Water Column (OWC) systems harness wave energy using a partially submerged chamber with an underwater opening. The Savonius turbine, a vertical-axis wind turbine, is well-suited for this purpose due to its efficiency at low speeds and self-starting capability, making it an ideal power take-off (PTO) mechanism in OWC systems. This study tested an OWC device with a Savonius turbine in an air duct to evaluate its performance under varying flow directions and loads. An innovative aspect was assessing the influence of power augmenters (PAs) positioned upstream and downstream of the turbine. The experimental setup included load cells, Pitot tubes, differential pressure sensors and rotational speed sensors. Data obtained were used to calculate pressure differentials across the turbine and torque. The primary goal of using PA is to increase the *C_P_–λ* curve area without modifying the turbine geometry, potentially enabling interventions on existing turbines without rotor dismantling. Additionally, another novelty is the implementation of a regression Machine-Learning algorithm based on decision trees to analyze the influence of various features on predicting pressure differences, thereby broadening the scope for further testing beyond physical experimentation.

## 1. Introduction

### 1.1. Overview

Energy and environmental challenges stand as major issues that humanity faces in the 21st century [1]. The escalating demand for energy, propelled by population growth and economic development, has led to a surge in greenhouse gas emissions, climate change, environmental degradation, and the depletion of natural resources.

At the forefront of global challenges lie climate change, resource scarcity—particularly in terms of energy—and environmental pollution. The former is inextricably linked to the surge in greenhouse gas emissions, predominantly driven by the widespread utilization of fossil fuels [2,3,4,5,6], resulting in climate shifts such as rising sea levels, intensified weather events, and ecosystem disruptions. Moreover, the energy demand, currently heavily reliant on oil, coal, and natural gas, has precipitated a gradual depletion of resources [7,8]. Simultaneously, the use of fossil fuels has engendered significant pollution, detrimentally impacting the environment and human health. Addressing the environmental repercussions of energy consumption necessitates a fundamental shift in the energy landscape towards renewable energy sources, efficient energy carriers [9,10,11,12,13,14], and different manufacturing processes [15,16]. Optimizing energy use through enhanced energy efficiency in end-use applications plays a crucial role in mitigating adverse climate impacts. Another compelling strategy to reduce dependency on traditional energy sources and mitigate environmental harm is tapping into ocean energy, notably wave energy. Despite its initial high installation costs and relatively lower efficiency, wave energy extraction presents a promising avenue for energy supply.

Among various wave energy conversion technologies, Oscillating Water Column (OWC) systems have emerged as particularly promising options [17,18]. These systems comprise a partially submerged chamber with an underwater opening on its front side and an air turbine. When waves interact with the device, the water column within the chamber undergoes oscillations, thus earning the system its name. These oscillations act akin to a piston, causing airflow alternation between exiting and entering the chamber’s upper section. This cyclic airflow propels the turbine, thereby generating power.

The Savonius turbine can serve as an effective power take-off (PTO) mechanism in OWC systems for efficient wave energy harnessing at a low cost [19,20]. The Savonius turbine, a type of vertical-axis wind turbine, has several attributes that make it particularly well-suited for integration with OWC systems. Firstly, the Savonius turbine operates effectively at low speeds and is capable of self-starting, which is beneficial given the variable and often low-speed nature of wave-induced flows in OWC systems. Its design, which consists of semi-cylindrical blades, allows it to capture energy from both the upward and downward motion of the water column, making it ideal for the bi-directional flow conditions typical of OWC systems. Additionally, the Savonius turbine is known for its simplicity and robustness. It has fewer moving parts compared to other turbine designs, leading to lower maintenance requirements and increased reliability, which are crucial factors for offshore energy systems where maintenance can be challenging and costly. This simplicity also translates to lower manufacturing costs, making the overall system more cost-effective. Moreover, the Savonius turbine’s ability to handle turbulent and irregular flows, which are common in wave environments, further enhances its suitability for OWC systems. Its vertical-axis orientation means it does not need to be reoriented to face the direction of the flow, unlike horizontal-axis turbines, thus continuously capturing energy regardless of changes in wave direction [21,22,23].

### 1.2. Literary Review

In two previous studies, the authors already investigated the topic of this work. The first one, [24], analyzes the fluid dynamics of a ducted Savonius wind turbine for OWC Wave Energy Converters. Using a 2D Computational Fluid Dynamics (CFD) [25,26,27] model validated against experimental data, the research demonstrates the turbine’s potential in OWC systems. Results indicate that the ducted Savonius rotor, with blockage effects considered, achieves a significantly higher power coefficient than bare Savonius turbines. The study suggests promising applications in mini and micro-generation systems, emphasizing the importance of steady unidirectional flow analysis for design optimization. In the second one [20], the performance of ducted Savonius turbines with a power booster in OWC systems was studied. Using converging sections with a Bell–Metha profile, the design aims to increase the mass flow rate and reduce vortices upstream of the turbine. A laboratory-scale Savonius turbine was tested with or without the power augmenter (PA) at a fixed air velocity. The results show that the power booster system enhances power output at all tip speed ratios, with maximum power at a flow oscillation frequency of 1 Hz. The increase in power is attributed to higher airflow velocity and improved pressure difference due to the duct contraction, although this also reduces the turbine’s power coefficient. Overall, the power augmentation method leads to a 10–20% average power increase, with peaks over 40%.

The study of Prasad et al. [28] examined the performance of Savonius rotors in OWC devices under various conditions, including wave characteristics, rotor geometry, water depth, submergence depth, and rotor/OWC orientation. A double OWC model was also tested. Key findings indicate that the optimal rotor configuration and water depth enhance performance, with specific orientations of the rotors yielding better results depending on the OWC setup. Increasing the blockage ratio did not improve performance, while the double OWC showed the best-combined rotor performance at certain frequencies. These insights help optimize Savonius rotors for efficient use in OWC systems. Dos Santos et al. [29] developed a computational model to investigate turbulent flows in OWC devices with a Savonius turbine. Three configurations were tested: a free turbine, an enclosed domain with constant inlet velocity, and an enclosed domain with sinusoidal inlet velocity. The model, validated against existing literature, successfully predicted power coefficients. Results showed increased power coefficients in the enclosed domain and similar performance between constant and sinusoidal velocities. The study highlights the importance of geometric configuration in turbine performance and suggests further research to include more realistic sea conditions and wave interactions.

The work of Prasad et al. [30] discussed the potential of wave energy, emphasizing the effectiveness of OWC converters. Traditional OWC designs face flow separation issues, prompting researchers to propose inclining the chamber. Numerical studies show that a 55° inclined OWC outperforms current and conventional designs, achieving a 13% higher maximum power at mean wave conditions and a peak power of 23.2 kW with improved efficiency.

Santos et al. described [31] the development of a computational model for simulating an OWC device with a Savonius turbine inserted in the inlet/outlet duct. The model couples the simulation of a two-phase, turbulent airflow with the turbine movement. Validation was conducted for both free stream turbulent flow over a Savonius turbine and wave flow over a converter without the turbine. The results demonstrated an increased power coefficient with the inserted turbine, indicating enhanced power takeoff due to the fairing of the turbine.

In the work of Zullah et al., the performance of Savonius-type turbines in OWC systems for wave energy conversion were investigated. Using numerical simulations, the study explored conventional and helical Savonius rotors. The results suggested that the helical rotor with twisted blades offers smoother operation, higher efficiency, and self-starting capability compared to conventional designs. The twisted blades increased the net positive torque, improving performance and indicating the potential for enhanced wave energy capture in OWC systems.

The paper of Dorrel et al. [32] presented a small segmented OWC with cascaded Savonius rotors and discussed modeling techniques for simulating its performance. Designed for shoreline locations like harbor walls, where waves are random, it achieves conversion rates of around 20% with a peak output of 25 W. The paper outlined a full algorithm solved using the Runge–Kutta–Nystrom method and validated the system’s operation through experimental tests. The results demonstrated good overall performance for small-scale applications, suggesting suitability for low-grade wave environments.

The work of Ciappi et al. [33] presented an analytical wave-to-wire model for optimizing OWC devices in the Mediterranean. The model integrated the chamber, air turbine, and electric generator. The study examined Wells and axial impulse turbines, finding that impulse turbines are more efficient and suitable for the location.

The paper of Henriques et al. [34] compared the performance of Wells and biradial turbines in OWC systems, focusing on the Mutriku wave power plant in Spain. It developed hydrodynamic and time-domain wave-to-wire models and highlighted the sensitivity of a Wells turbine performance to control parameters. The biradial turbine exhibited higher efficiency and can operate closer to maximum efficiency over a broad range of control parameters, showing a 32% higher annual-averaged efficiency compared to the Wells turbine at the Mutriku power plant. The study emphasized selecting control laws based on generator efficiency and suggested statistical analysis for optimal performance.

About the application of ML algorithms for a predictive model, the study of Seo et al. [35] explored the application of machine learning-based prediction technology, as an element of digital twin technology, to predict pressure in the OWC of a wave power plant. By analyzing correlations between wave height data and OWC sensor data, meaningful parameters for prediction were identified. Feature engineering was employed to extract relevant features from the dataset, and a high-performing machine-learning model was selected after training with various models. The study demonstrated the potential for improved operational efficiency by predicting OWC pressure, which is critical for wave energy conversion.

Seo et al. [36] explored the application of ML for improving the efficiency of wave power plants by predicting the pressure inside the OWC chamber. Using correlations between wave height and sensor data, a training model based on a digital twin of an OWC was designed. ML models were employed to predict chamber pressure, with promising results. Despite data limitations, the study highlighted the potential of ML in enhancing wave power plant operation efficiency, suggesting future research directions for improved accuracy and real-time data processing.

The work of Roh and Kim [37] applied deep learning algorithms to predict turbine generator rotational speed in OWC wave energy converters for improved control. By preemptively operating valves based on predicted speeds, the energy input can be managed more effectively. Various deep learning algorithms were compared using operational data from an OWC wave energy converter off the coast of Jeju, South Korea, with LSTM showing the most accurate predictions. This research highlighted the potential of deep learning for enhancing OWC operational efficiency.

The study of Marques-Silva et al. [38] focused on short-term wave forecasting to enhance the economic viability of OWC, particularly those equipped with biradial turbines. Using data from the Mutriku wave power plant in Spain, three regression algorithms—LS-SVM, AR, and ARMA—were developed to predict air chamber pressure. LS-SVM models with fewer features demonstrated good performance with average errors of 19%, while AR and ARMA models showed similar performance with average errors of around 18%. However, LS-SVM models suffered from increased errors when extending the forecasting horizon due to limited training data. On the other hand, the AR and ARMA models exhibited consistent performance across different forecast horizons. Considering computational efficiency, ARMA emerged as the preferable option for model predictive control strategies in real wave power plants.

### 1.3. Aim of the Work

The main purpose of the study is to test a device that can enhance the performance characteristics of the Savonius turbine in terms of *C_p_–λ* (these parameters will be discussed later). The device should be easy to implement so that it can also be applied to Savonius turbines already in operation. Additionally, thanks to the numerous experimental tests conducted (1044), the study has enabled the development of a regression ML algorithm based on a decision tree that can be used to predict the turbine’s behavior beyond the laboratory-tested boundary conditions. Specifically, experimental tests were made subjecting the turbine to a mono-directional or bi-directional airflow with varying load conditions, and the data were processed using ML to extract the factors influencing turbine performance. The utilization of ML techniques allowed for a comprehensive evaluation of various parameters affecting turbine efficiency. The outcome of this evaluation can be used to set an optimization process to enhance performance or efficiency as it is already being done in different engineering fields [24,39,40,41]. The performance of the turbine can be influenced by deflectors positioned upwind or downwind of the turbine [24,42,43,44].

In this research, the Bell–Metha polynomial law was used to design PAs that act as convergent ducts. These augmenters can be strategically placed both upwind and downwind of the turbine to enhance its performance. By employing this approach, the rotational speed of the turbine increases, thereby influencing its overall efficiency.

ML algorithms based on decision trees were subsequently implemented to extract insights into the parameters exerting the most significant influence on turbine power production. Furthermore, the utilization of an ML regression model enables the exploration of diverse configurations of the used parameters without necessitating alterations to the setup. The advantage of applying this ML algorithm is the ability to predict the value of the pressure drop (between the upwind and downwind size of the turbine) generated through new configurations without the use of sensors and to vary the configuration of the real system. Therefore, what can be defined as a partial digital twin of the system is obtained, with which it is possible to test different combinations of parameters to achieve the best performance.

## 2. Materials and Methods

### 2.1. Experimental Setup

**Figure 1:**
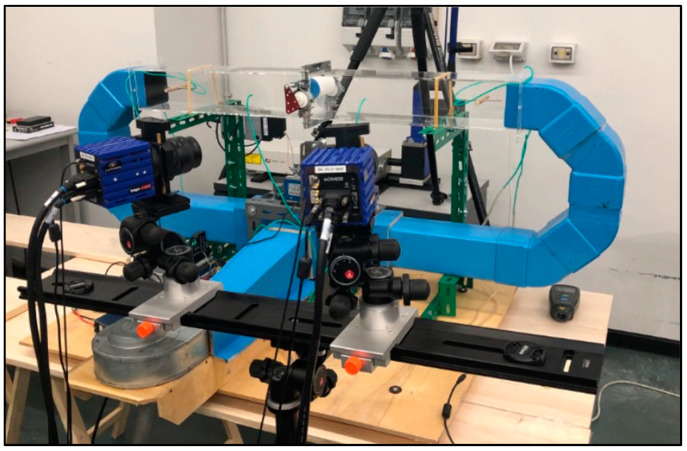
Test tube in the UNIME lab.

The experimental tests were conducted in a test tube at the University of Messina (Figure 1). The upper portion of the test tube is constructed with PVC walls, forming an internal square cross-section measuring 100 × 100 mm and extending 1000 mm in length. The turbine is positioned at the midpoint of the upper section. The other parts of the setup are made of wood and PVC.

The lateral regions comprise two 45° bends (a single bend is 160 mm long) connected by a vertical junction, also with square cross-sections. In the lower section, there is a fan that generates the airflow and a diverter. The diverter can rotate at a preset frequency *f*, enabling the redirection of airflow towards one of the two curves. When *f* = 0 the flow is mono-directional; when *f* > 0 the flow is bi-directional, and the inversion velocity can be controlled.

**Figure 2:**
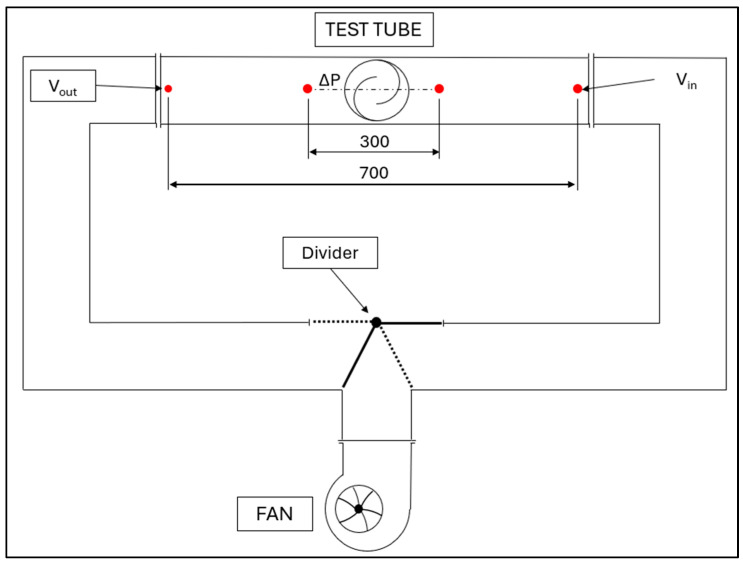
Layout of the test tube, dimensions in mm.

The layout of the test tube is presented in Figure 2.

**Figure 3:**
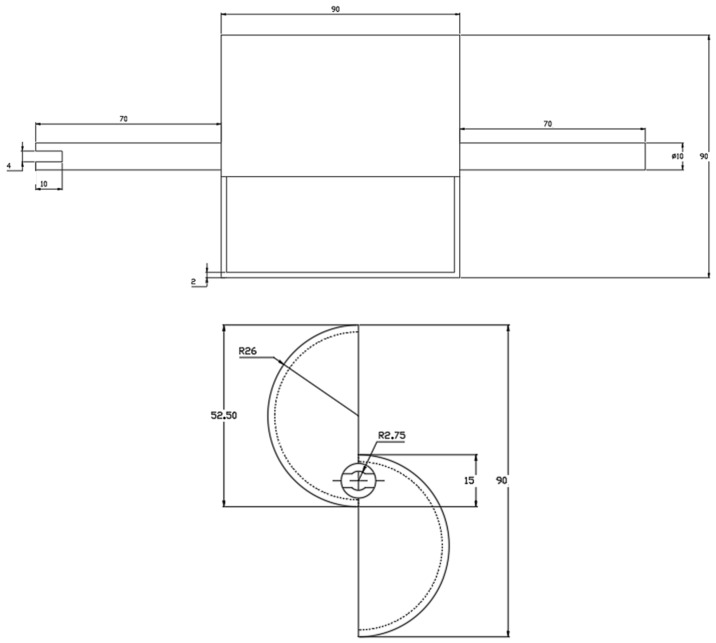
Dimensions of the Savonius turbine.

The turbine (Figure 3) was 3D-printed in PLA. The principal characteristics are listed in Table 1.

**Figure 4:**
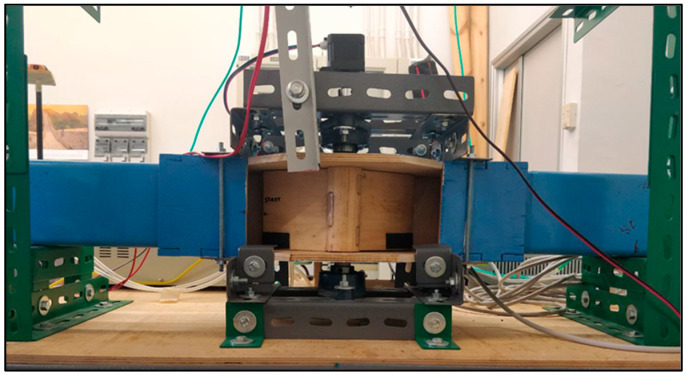
Mobile diverter.

The airflow is generated by a centrifugal fan, the EBM Papst G1G133-DE19-15, powered by a 24-volt DC voltage. This fan operates at a rotational speed of 2000 r/min and has an outlet section measuring 59 × 71 mm. In the present study, both mono-directional and bi-directional tests were conducted. For this purpose, the diverter swings around its hinge, directing the airflow from the fan towards one of the lateral sections depending on its position. The diverter is actuated by a stepper motor and is presented in Figure 4. The bi-directional flow simulates the rising and falling of waves in the sea.

The operating conditions of the flow and the dimensions of the turbine are constrained by the limits of the experimental setup related to the test chamber. The input power is limited by the fan’s power, and the turbine’s dimensions are restricted by the chamber’s geometry. In order to explore and design the characteristic curve *C_P_–λ*, since the rotational speed of the fan is fixed, the turbine was slowed down adding masses to the free end of a belt brake. In doing so, even if the airflow speed does not change, *λ* values vary because the rotational speed of the turbine is changing (see Equation (14)).

The process from power input to energy production involves three main stages: the first stage pertains to the power input (in this case, the fan), the second stage involves converting the primary energy into mechanical energy, and the final stage concerns converting mechanical energy into electrical energy. The power input, as defined during the test, is constrained by the fan’s power, so this primary energy remains constant for each configuration tested. Therefore, comparisons among all proposed configurations must be conducted with the same input power.

Another important aspect is the conversion of the energy produced by the turbine into electrical energy using an electric generator. In the experimental tests conducted, this control strategy is not considered because the study’s main goal is to use a device (power augmenter) to increase the performance of the Savonius turbine under the same boundary conditions. The stages upstream and downstream of the Savonius turbine are maintained and considered constant. The primary and tertiary energy conversion stages are considered not important at this stage because they have the same effects on the turbine with 1, 2, or without PAs.

#### 2.1.1. Power Augmenters (PAs)

**Figure 5:**
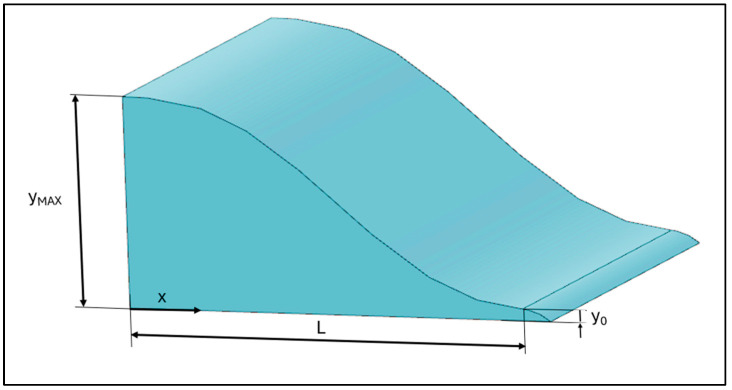
Power augmenter dimensions.

The aim of the PA is to create a converging nozzle in front of the active blade of the Savonius turbine, as the converging nozzle helps to increase the velocity. Additionally, the power augmenter masks the part of the turbine that would generate resistance to rotation (as shown in Figure 5). The shape of the power augmenter was chosen to be easily manufactured (with a constant shape along the z-direction) and to comply with the geometric constraints imposed by the test chamber on the turbine. There are several shapes that can be used as power augmenters, such as Spline, NURBS, Bezier Curves, and Polynomial curves. For this phase, a polynomial curve was chosen for the section profile of the PA. Generally, increasing the degree of a polynomial function allows for more geometric parameters to be managed. However, this also introduces many unknowns, which could pose certain limitations from an optimization perspective using numerical methods. A good compromise is a 5th-degree polynomial, such as the Bell–Metha polynomial. The Bell–Metha equation allows control over various geometric aspects of the profile, including not only the maximum height of the profile, constrained by the dimensions of the turbine, but also the position of the inflection point of the curve. The equation of the polynomial is provided in (1).

(1) $$y=a\xi^{5}+b\xi^{4}+c\xi^{3}+d\xi^{2}+e\xi +f$$

y is the height of the PA, x a generic length expressed in millimeters, L the total length of the PA and ξ the dependent variable defined as *x*/*L* (Figure 5).

**Figure 6:**
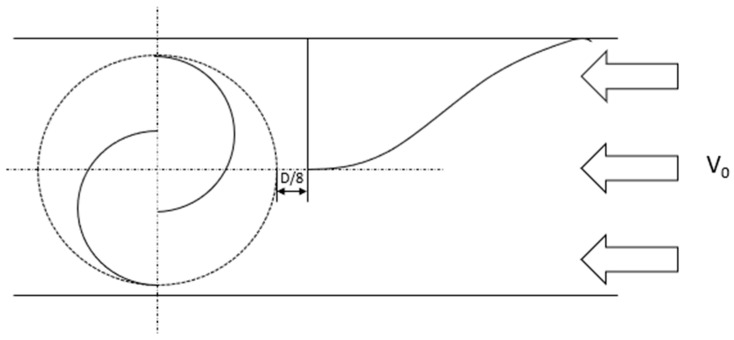
Example of upwind power augmenter positioning (*f* = 0 Hz).

The experimental tests conducted help to understand the effects of the presence of the PA and, in general, help to evaluate the influence of the distance of the power augmenter. The distance influences the PA’s efficiency because the turbulence behind the power augmenter can have positive or negative effects depending on its distance from the active part of the turbine. Depending on the frequency of the diverter, the turbine can be tested both in mono and bi-directional flow conditions. Different distances between the turbine and the power augmenters were tried. In mono-directional test cases, only the upwind power augmenter was placed since the flow has only one clear direction; in the bi-directional tests, the power augmenters were placed both upwind and downwind. In Figure 6, the positioning of the power augmenter with a distance of *D*/8 in a mono-directional test is represented.

#### 2.1.2. Measurement Devices

The measurements are made through two velocity sensors, a load cell, and two pressure sensors. The analogic board used to process signals from the sensors is the NI cDAQ 9171 (National Instruments, Austin, Texas, USA). These signals are processed and managed using the software LabView on the computer.

The diverter is rotated using a stepper motor and it is operated via an Arduino code, which allows setting the desired rotation frequency.

**Figure 7:**
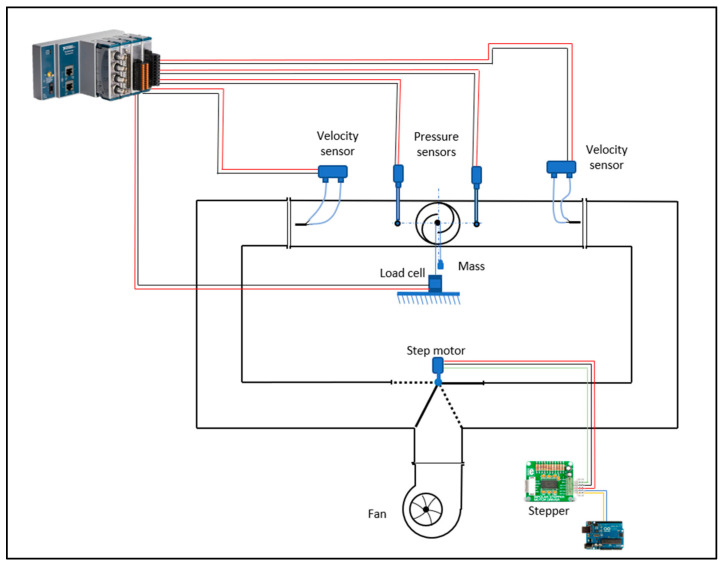
Connections between measurement devices and control systems.

The connections between all the devices are represented in Figure 7.

##### Torque Measurement

**Figure 8:**
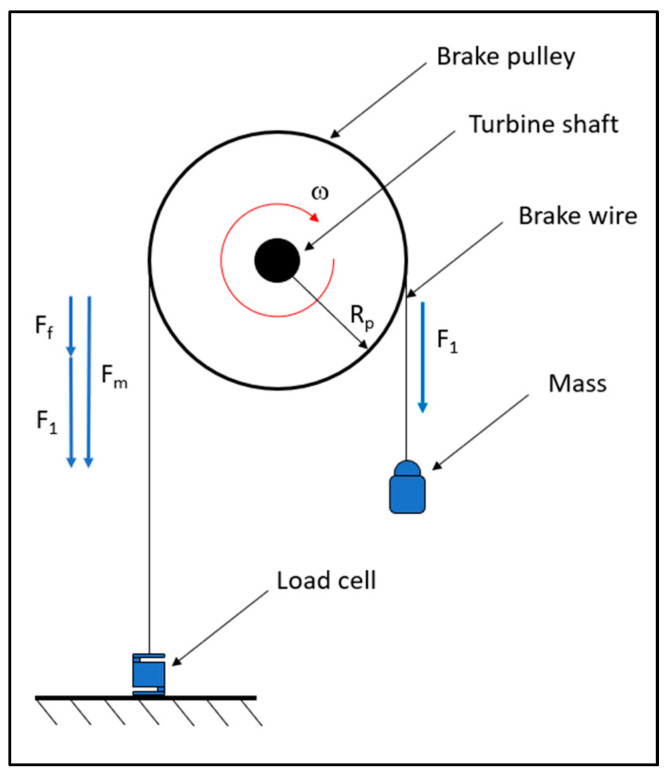
Torque measurement system.

To measure the torque developed by the turbine, a belt brake composed of a pulley, a nylon wire, a suspended mass, and a load cell was employed (Figure 8).

The nylon wire is connected at one end to the load cell fixed to the ground, runs along the pulley, and at the other end, there is a hook with which it is possible to suspend a mass. Since the pulley is keyed to the turbine shaft, its rotation speed varies depending on the suspended mass. Figure 8 shows a schematic of the system used and the balance of dynamic forces.

- $F_{1}$: weight force;
- $F_{f}$: friction force acting on the pulley;
- $F_{m}$: force measured by the load cell.

Under static conditions, since $F_{f}=0$:

(2) $$F_{m}=F_{1}$$

However, under dynamic conditions, the force measured by the cell will be:

(3) $$F_{m}=F_{1}+F_{f}$$

The torque *T*, expressed in N·m, can be therefore calculated:

(4) $$T=F_{m}\cdot D_{pulley}/2$$

##### Pitot Tubes

To calculate the fluid velocity inside the test tube, two Pitot tubes were placed, each at 100 mm from the two bends of the upper section. These are two tubes immersed in the fluid, one facing upstream and the other downstream. Both are equipped with a hole at the front that serves as a total pressure (or stagnation pressure) port. Additionally, longitudinally, at a certain distance, there are additional holes acting as static pressure ports.

The relationship between pressure and velocity is expressed by Bernoulli equation.

(5) $$P_{st}+\frac{1}{2}\rho V^{2}=P_{tot}$$

(6) $$V=\sqrt{\frac{2\left(P_{tot}-P_{st}\right)}{\rho }}=\sqrt{\frac{2∆P}{\rho }}$$

The data collected from the Pitot tubes and the pressure taps upstream and downstream of the rotor (Δ*P*) were processed by a pressure transmitter. Three HD420T (DeltaOhm, Caselle, Italy) devices were used: two for the Pitot tubes and one to determine the pressure drop near the turbine.

##### Rotary Encoder

To measure the turbine rotational speed, a rotary encoder was used. Positioned in front of a ring with alternating black and white bands, this device produces a voltage corresponding to the band aligned with the sensor. The resulting signal is a square wave.

Each time the encoder detects a white band, it generates a peak in the signal. The pulses per second (*PPS*) can be calculated using Equation (5).

(7) $$PPS=\frac{PPS_{tot}}{T}$$

$PPS_{tot}$ are the total pulses recorded during the test and T the recording period in seconds.

Knowing that there are 8 white bands in the encoder, once the pulses per second are calculated, the rotational speed in r/min of the turbine can be calculated.

(8) $$n=\frac{PPS}{8}\cdot 60$$

##### Data Acquisition

Data collection is divided into two phases: one with the fan turned on and one with the fan turned off. By comparing the data from these two phases, it is possible to evaluate the turbine’s torque and effective power since in the second phase, the friction force on the pulley, $F_{a}$ is zero.

The software used to record the tests is NI LabVIEW(v. 2018). A recording duration of 20 s was set with a sampling frequency of 0.001 s. The force detected by the load cell, the pressure drop measured by the Pitot tubes and the encoder signal in volts were acquired.

Furthermore, to ensure accurate analysis, it was necessary to reset the load cell so that it did not consider the mass of the screw, nylon wire, and hook connected to it, thus evaluating the torque without taking external elements into account.

Since each acquisition has 20,000 data points, it is necessary to calculate the mean of all values obtained to determine the mean value of the airflow velocity $V_{0}$, the mean value of ∆P between upstream and downstream of the turbine, and the mean value of the applied load L.

In particular, the airflow velocity and the applied load can be obtained using Equations (8) and (9).

(9) $$V_{0}=\sqrt{2\cdot \frac{∆P_{Pitot}}{\rho }}$$

(10) $$L=\frac{1}{n_{data}}\cdot \left(\sum Load_{ON}-Load_{OFF}\right)$$

with:

$n_{data}$: number of registered data;

$Load_{ON}:$ load measured with the fan on;

$Load_{OFF}:$ load measured with the fan off;

The power *W* of the turbine and the available power *W_in_* are expressed in Watt.

(11) $$W=2\pi \cdot \frac{n}{60}\cdot T$$

(12) $$W_{in}=\left(\frac{1}{2}\cdot \rho \cdot A_{turbine}\cdot V_{0}^{3}\right)+\left(∆P\cdot A_{turbine}\cdot V_{0}\right)$$

Once these parameters are obtained, the value of the power coefficient $C_{p}$ and the tip speed ratio λ are determined.

(13) $$C_{p}=\frac{W}{W_{in}}$$

(14) $$\lambda =\frac{2\pi \cdot n\cdot D_{turbine}}{120\cdot V_{0}}$$

The measurements were averaged over the acquisition period, so all the considered performance coefficients are not instantaneous.

#### 2.1.3. Tested Configurations

Different configurations were tested, varying the number and the positioning of the Power Augmenters (PAs). A complete summary of the conducted tests is presented in Table 2.

The test on a certain configuration is repeated at least five times, to check and ensure the repeatability of the measurement.

### 2.2. Machine Learning

Machine Learning (ML) [45,46] is a branch of artificial intelligence (AI). It originated in the 1950s in Hanover [46] and focuses on systems that perform tasks that, to an external observer, would appear to be exclusively within the domain of human intelligence. ML emerged as a subset of AI following various schools of thought that defined an “intelligent” system as one capable of learning from experience and improving its performance [47]. This distinctive trait sets ML algorithms apart from conventional computer programming, as they can operate even under conditions for which they have not been explicitly programmed.

The main objective of testing the physical model with different configurations (frequency, load, number of power augmenters, and power augmenter distance) is to maximize the power output of the turbine. With the same input power (fan), the aim is to maximize the output power (generated by the turbine). As mentioned earlier, one of the fluid parameters measured by dedicated sensors during the various tests is the pressure difference (Δ*P*) created between the upstream and downstream stages of the turbine. The investigation aimed to identify which parameters, when varied within the different system configurations, had the greatest impact on the generation of Δ*P*. Understanding which parameters most significantly influence the value of Δ*P* would allow for coherent modification of the setup to maximize the turbine power output. To recap, the parameters varied during the different tests are applied load, presence/absence of power augmenters, number of power augmenters, flow feed frequency, and distance between the power augmenters. Among these parameters, the presence/absence of power augmenters and the number of power augmenters are categorical variables, unlike all the others which are continuous variables. Table 3 summarizes the setup variables along with a description and range of variation.

The Δ*P* is evidently a continuous quantity with a variability range of [−151.9606, 85.0614] Pa. This range of Δ*P* is determined by computing the pressure difference between measurements taken downstream and upstream of the turbine. Thus, the negative sign denotes instances where the downstream pressure surpasses that measured upstream of the turbine (with consideration for potential variations in fluid flow direction). However, for power generation considerations, the absolute value of ΔP is paramount, given the self-aligning property of the Savonius turbine, which rotates unidirectionally regardless of fluid flow direction. Therefore, Δ*P* is evaluated in absolute terms, as power production remains unaffected by fluid flow direction.

With the available dataset, derived from the measurements taken, it was decided to train a ML algorithm for two main reasons:

- To identify the parameters that most influence Δ*P*;
- To obtain a model capable of hypothesizing new scenarios without the constraint of necessarily intervening in the physical system.

As is well known, an ML algorithm is capable of learning a relationship between the features and the output, even under conditions it has never encountered before, unlike in traditional programming.

The dataset consists of 5 input variables (referred to as “features”) and one output variable (Δ*P*), comprising 1044 instances, or observations. This dataset was then split to obtain three distinct datasets, each for one of the three phases required to develop a robust ML model: training, validation, and testing. Approximately 10% of the dataset (100 instances selected randomly) was reserved for the testing phase, while the remaining data underwent a 10-fold cross-validation. Table 4 provides an overview of the datasets and the number of instances they contain.

This practice is employed to enhance the model’s performance, especially when the dataset is not extensive. Consequently, the remaining 90% of the dataset is divided into 9 sections. Ten training and validation sessions are conducted, using 9 sections for training and 1 section for validation during each session.

In each phase, a different section will be used for validation. During validation, the model’s performance is evaluated and improved. The unique aspect of using cross-validation is that it not only estimates the performance of the trained model but also provides a measure of how accurate its predictions are (through evaluation of the standard deviation) and how reproducible they are. Finally, the test dataset simulates a real application of the model, aiming to observe its behavior with data it has never seen during training. The test results provide a measure of the model’s goodness.

The choice of the ML model to implement fell on decision trees, which are a predictive model based on a tree structure for decision-making. The advantages of this model for the problem addressed in this work will be discussed later.

A decision tree model is a structure composed of nodes and leaves. Each node represents a question or condition about a data attribute, and each leaf represents a class or output value, depending on whether it is a classification or regression problem.

**Figure 9:**
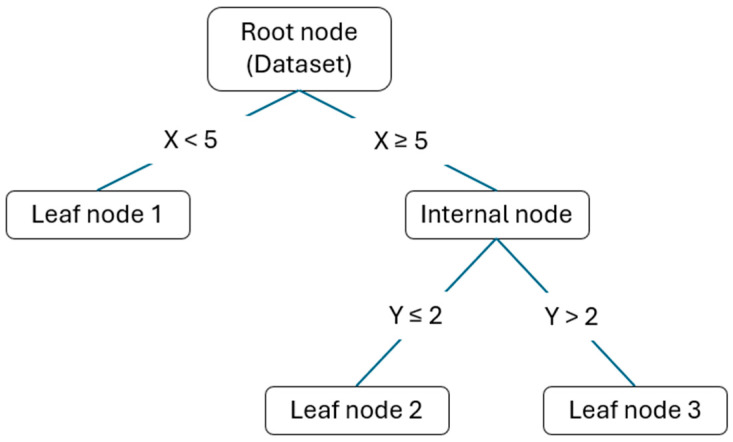
Schematization of a decision tree.

At the beginning of the tree is the root node, which contains the entire training dataset. From here, the tree branches into child nodes, each of which represents a possible response to the question or condition posed by the parent node. Each child node is connected to the parent node by a branch, indicating the hierarchical relationship between them. When descending along the decision tree, encounters with intermediate nodes occur, which continue to pose questions or conditions on the data based on previous answers. This process continues until it reaches the tree’s leaves, where a final decision is made, or a predictive output is provided. Figure 9 shows a schematization of a decision tree.

The algorithm underlying the operation of decision trees is called Classification and Regression Tree (*CART*). It is used for both classification and regression problems. The problem addressed in this work falls within the realm of regression. Indeed, the output variable is a continuous variable (unlike a classification problem where the output is a class). Therefore, in this case, the model will predict continuous values, and to assess the model’s performance, the error in estimating these values is measured.

*CART* constructs a decision tree by splitting the dataset into two subsets of data according to a feature (*k*) and a certain threshold (*t*). The feature is chosen by carefully evaluating the *k*-*t* pairs that minimize a certain function. In the case of regression, the goal is not to determine a class but to obtain a value. The cost function to be minimized is based on the Mean Square Error (*MSE*) and is as follows [48]:

(15) $$J\left(k,t_{k}\right)=\frac{m_{left}}{m}MSE_{left}+\frac{m_{right}}{m}MSE_{right}\;with\;\;\left\{\begin{array}{c} MSE_{node}=\sum_{i\;\in \;node}{\left({\hat{y}}_{node}-y^{\left(i\right)}\right)}^{2}\\ {\hat{y}}_{node}=\frac{1}{m_{node}}\;\sum_{i\;\in \;node}y^{\left(i\right)} \end{array}\right.$$

where:

- *MSE* is calculated as the average of the squares of the differences between the values predicted by the tree (${\hat{y}}_{node}$) and the corresponding actual values in the training data ($y^{\left(i\right)}$). Minimizing the *MSE* during tree construction helps find optimal splits that reduce the overall prediction error;
- m is the total number of instances in the training dataset;
- $m_{node}$ represents the number of instances contained in the node of interest;
- The number of instances to the right ($m_{right}$) and left ($m_{left}$) refers to the number of training samples ending up in the right and left subtree, respectively, during the tree-splitting process.

This aspect is crucial because during the tree construction phase, the goal is to find splits that minimize predictive error while simultaneously avoiding overfitting. Therefore, the splits must be chosen to minimize the *MSE* and ensure that each subtree has a sufficient number of instances to make accurate predictions.

Overfitting occurs when a model excessively fits the training data, capturing the noise present in the data rather than just the relevant patterns. In this way, the decision tree runs the risk of becoming too complex, with many splits and nodes, to the point of effectively memorizing the training data instead of being able to generalize correctly to new, unseen data. As a result, the model may fail to generalize, meaning it cannot properly evaluate new data, as it has overfit to the training data.

The choice of a decision tree-based ML algorithm to address the problem proposed in this work is supported by a series of motivations outlined below.

This ML model is capable of working with mixed variables (continuous and categorical) without needing to convert categorical ones into binaries through a process called one-hot encoding. Moreover, they can achieve good performance even with relatively small datasets (on the order of thousands of data points as in the case of the dataset used in this work). The computational burden required for training a decision tree model is very low since they are quickly implementable and do not require data normalization. The most important characteristic of decision trees, for the purposes of this work, is that they behave like white boxes. Unlike many other ML methods such as neural networks, which behave like black boxes, a decision tree model is highly interpretable. Therefore, it is possible to understand how the model makes its prediction. This allows for the identification of the most influential input variables on the output.

To analyze the model’s performance, that is, to obtain a quantitative value of the trained model’s goodness, various metrics are calculated using the results of the validation and test. In particular:

- *MSE*, as already defined, calculates the average of the squares of the differences between the model’s predicted values and the actual values in the validation (or test) dataset. This metric is particularly sensitive to outliers;
- Root Mean Square Error (*RMSE*) is the square root of the *MSE* and provides a measure of the average prediction error in units of the output variable. It corresponds to the Euclidean norm [48].

(16) $$RMSE\left(X,h\right)=\sqrt{\frac{1}{m}\sum_{i=1}^{m}{\left(h\left(x^{\left(i\right)}\right)-y^{\left(i\right)}\right)}^{2}}$$

where $h\left(x^{\left(i\right)}\right)$ is the predicted value.

- The coefficient R^2^, also known as the coefficient of determination, provides a measure of how well the model fits the data. R^2^ varies between 0 and 1 and represents the percentage of variation in the output variable explained by the model. A value closer to 1 indicates a better model fit;
- Mean Absolute Error (*MAE*) calculates the average of the absolute differences between the predicted values and the actual values and is less sensitive to outliers compared to *MSE* [48].

(17) $$MAE\left(X,h\right)=\frac{1}{m}\sum_{i=1}^{m}\left|h\left(x^{\left(i\right)}\right)-y^{\left(i\right)}\right|$$

The ML model was trained using the “Regression Learner” app of MATLAB (MathWorks, v. R2023b). The chosen algorithm type is “Optimizable Tree”, which explores different combinations of hyperparameters to achieve the best performance of the model.

Hyperparameters are configuration settings external to the model and cannot be directly estimated from data. They are set before the training process and govern the behavior of the learning algorithm. Unlike parameters, which are learned during training, hyperparameters are typically chosen based on heuristics, prior knowledge, or through a process of trial and error [49,50]. In particular, the hyperparameter used are the minimum leaf size, the maximum number of splits, and the minimum parent size. The minimum leaf size is the minimum number of observations per tree leaf. Smaller values may lead to more complex trees, potentially prone to overfitting. The maximum number of splits allowed in each tree. This parameter can control the complexity of the tree. The minimum number of observations per tree parent. Smaller values may lead to more splits and potentially more complex trees.

As regard the interpretation of the results with the aim of understanding the weight of each feature (or predictor) on the output, a prediction importance was conducted. The prediction importance is evaluated by looking at how much the risk of a node changes when a split is made based on that predictor. This change in risk is measured as the difference between the risk at the parent node and the combined risk at the two child nodes created by the split. For example, if a tree divides a parent node (like node 1) into two child nodes (nodes 2 and 3), the importance of the predictor involved in this split is boosted by:

(18) $$\left(R_{1}-R_{2}-R_{3}\right)=N_{branch}$$

where $R_{i}$ represents the node risk of node i, and $N_{branch}$ is the total number of branch nodes. Node risk is determined by multiplying the node probability ($P_{i}$) by the related *MSE*:

(19) $$R_{i}=P_{i}\;MSE_{i}$$

In which the node probability ($P_{i}$) proportion of observations in the original dataset that meet the conditions for each node in the tree.

## 3. Results and Discussions

### 3.1. Experimental Results

To characterize the turbine behavior, the *C_P_–λ* curves (see Equations (13) and (14)) were extracted from the experimental results. Each point of the curve is the average of five experimental results for that specific condition.

**Figure 10:**
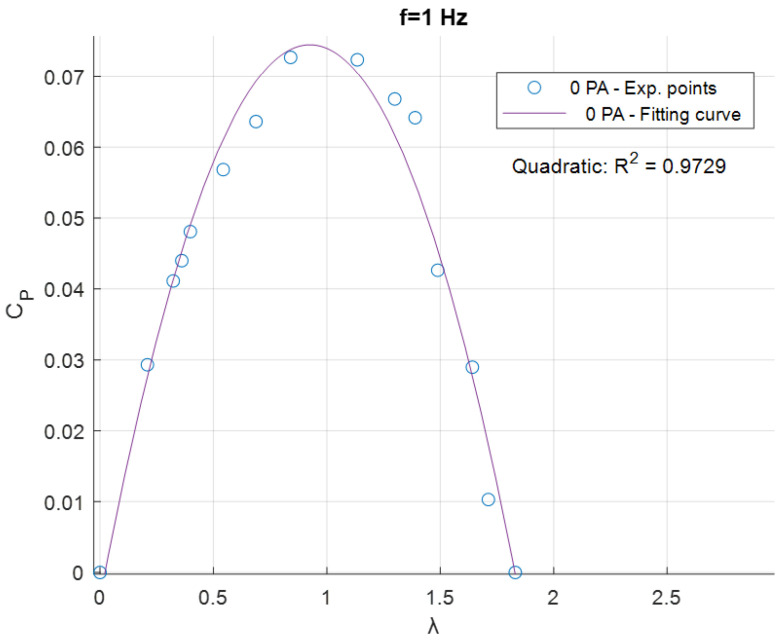
Example of experimental points curve fitting.

Since multiple results will be compared, for better clarity and visualization, the results points for every experimental test were fitted with a quadratic curve. In Figure 10, an example of fitting for the *f* = 1 Hz and 0 PA test is shown.

**Figure 11:**
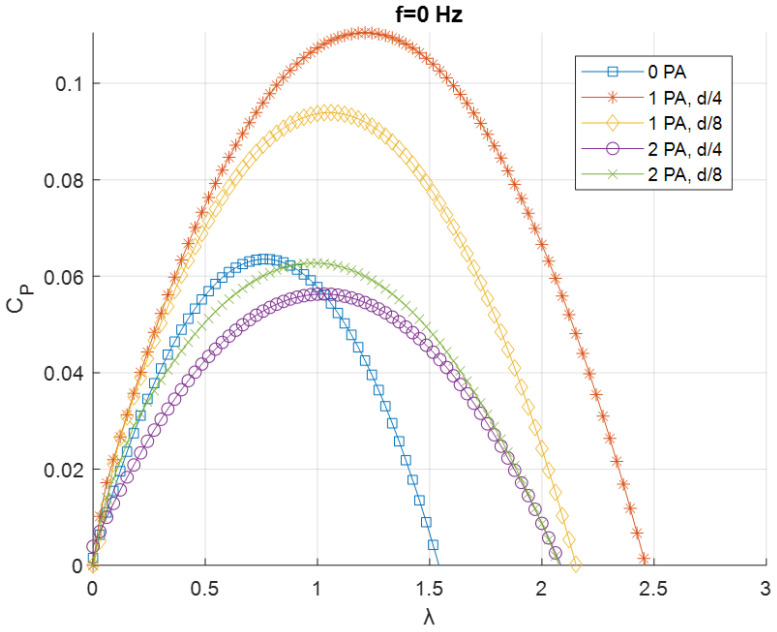
*C_P_–λ* curves for f = 0 Hz.

**Figure 12:**
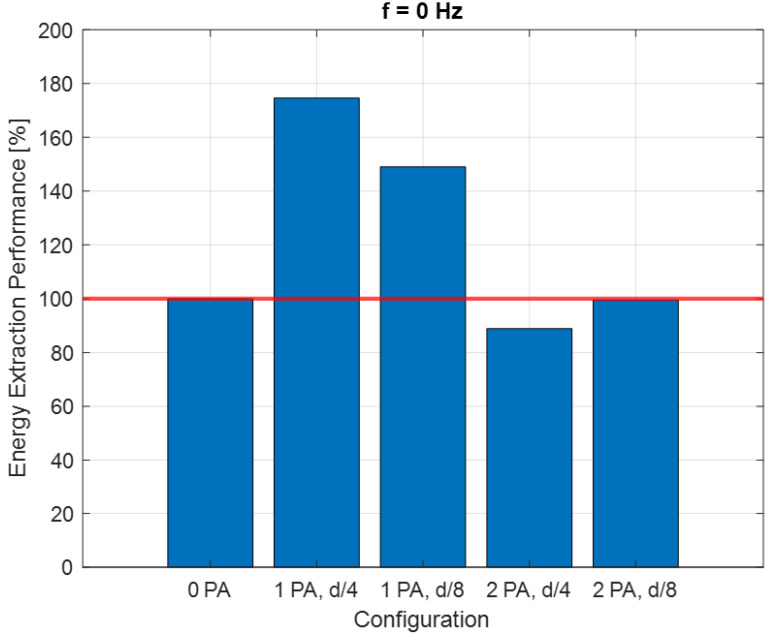
Energy extraction performance for 0 Hz configuration.

**Figure 13:**
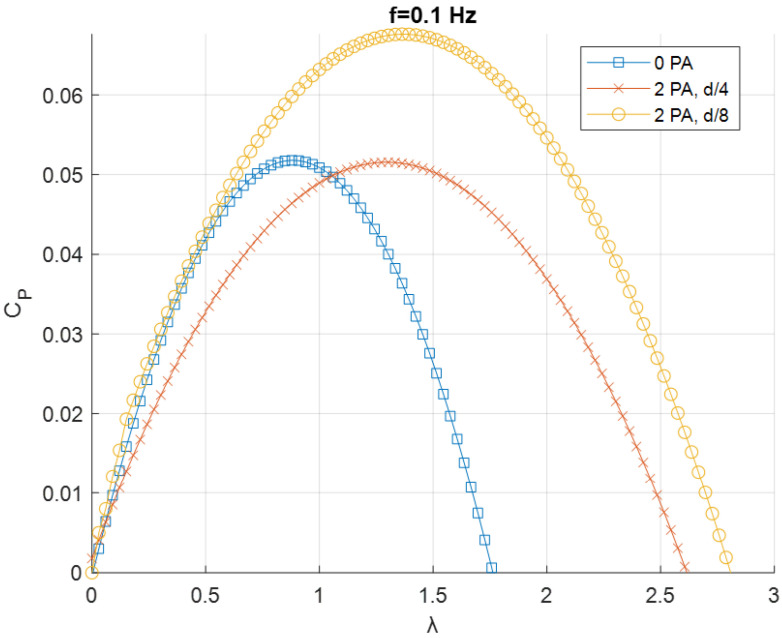
*C_P_–λ* curves for *f* = 0.1 Hz.

The resulting fitted curves were grouped based on the frequency *f* of the flow generated by the fan (Figure 11, Figure 12 and Figure 13).

The area under a *C_P_–λ* curve provides an estimate of the energy that can be recovered from the wave motion. This area can be increased either by speeding up the rotation of the turbine or by improving the power coefficient.

The energy that can be extracted by the turbine working at the maximum *C_P_* condition was considered for each configuration. An Energy Extraction Performance (*EEP*) index was introduced:

(20) $$EEP\;[\%]=\frac{W\left(CPmax,i\right)\cdot t}{W(CPmax,\;0\;PA)\cdot t}\cdot 100$$

*W*(*C_Pmax_*,*i*) is the power generated by the turbine for the generic “*i*” configuration with a certain PA number and positioning and with a rotational speed corresponding to the *C_Pmax_* point. *W*(*C_Pmax_*, 0 PA) is the power referred to in the configuration with no PA. The *EEP* index is recalculated for each of the three flow frequency values.

Figure 11 shows that with a mono-directional flow, as expected, the biggest improvement is given by the 1-PA configurations. An interesting effect is that by decreasing the distance from the blades, the effects in terms of general improvements are reduced. The 2-PA configurations do not give an improvement in terms of power, since the downwind PA disturbs the wake of the turbine generating turbulence. However, an increase in the tip speed ratio is registered due to the presence of the upwind PA.

As shown in Figure 12, in the case of the mono-directional flow, the presence of one power augmenter is very important in terms of energy extracted. It is clear that, for this particular situation, as mentioned above, the two power augmenters are counterproductive because there is no advantage in terms of energy recovered. It is fascinating to observe that the optimal solution occurs at a distance equal to *D*/4. Placing the PA too close results in a less significant effect.

Figure 13 shows the case for *f* = 0.1 Hz. When the flow is not mono-directional, like in this case and for *f* = 1 Hz, the condition with only one PA is not considered, since the flow would find a convergent duct in one direction only. Adding two PAs led to a sensible increase only in tip speed ratio for a distance of *D*/4. For a distance of *D*/8, a relevant increase in the power coefficient is registered, making this the best condition performance-wise among the tested ones.

**Figure 14:**
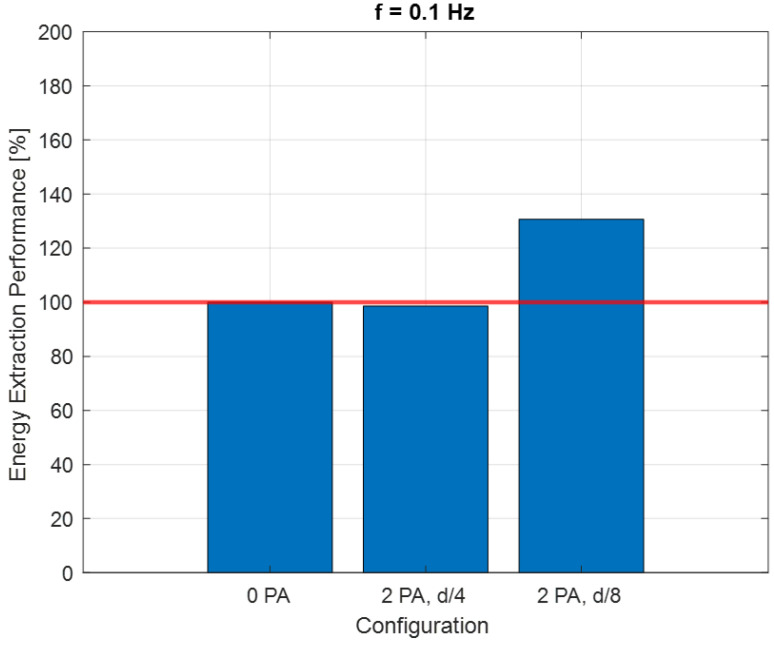
Energy extraction performance for 0.1 Hz configuration.

As shown in Figure 14, in the case of the bi-directional flow, it is very important to manage the distance between the PAs. There is a specific distance within which the effect of the PAs is positive; beyond this distance, the presence of the PAs has no effect.

**Figure 15:**
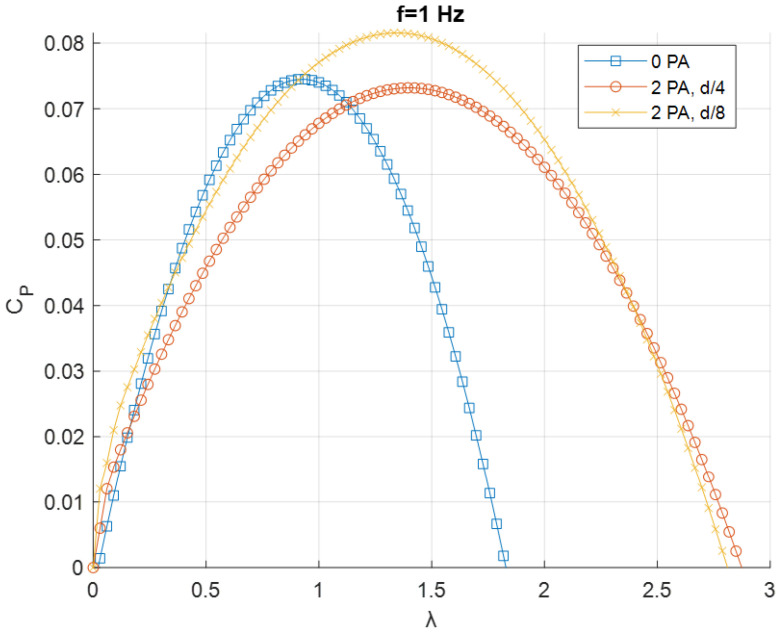
*C_P_–λ* curves for *f* = 0.1 Hz.

Figure 15 refers to the cases with *f* = 1 Hz. For this condition, as for *f* = 0.1 Hz, the best condition performance-wise is with two PAs at *D*/8 from the turbine. The increase in terms of power coefficient and tip speed ratio when referring to the case with no PA, however, is not as big as in the *f* = 0.1 Hz condition.

**Figure 16:**
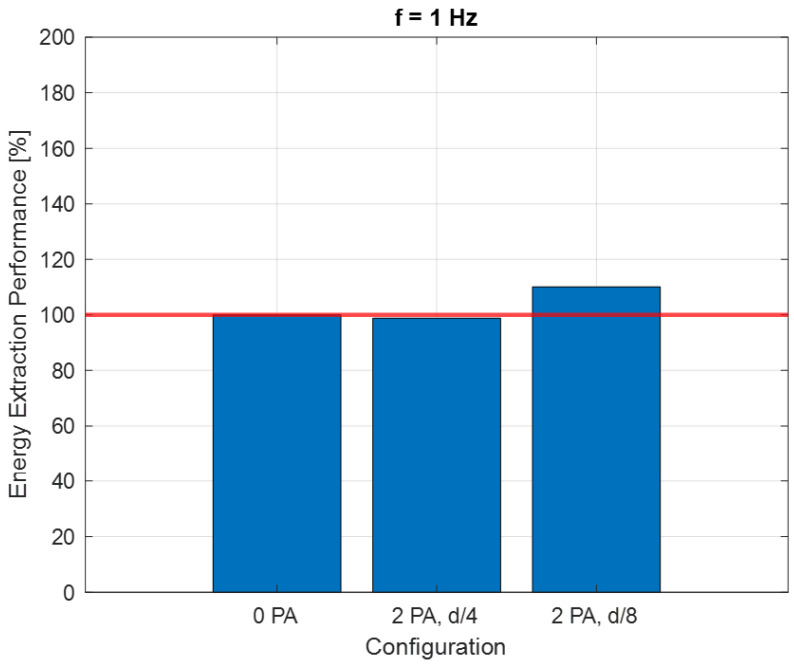
Energy extraction performance for 1 Hz configuration.

As shown in Figure 16, in the case of the bi-directional flow with a higher frequency, the effect of the distance is different and in this case, the *D*/4 configuration is negative with respect to the 0-PA configuration. The graph highlights the importance of the frequency in this kind of configuration.

**Figure 17:**
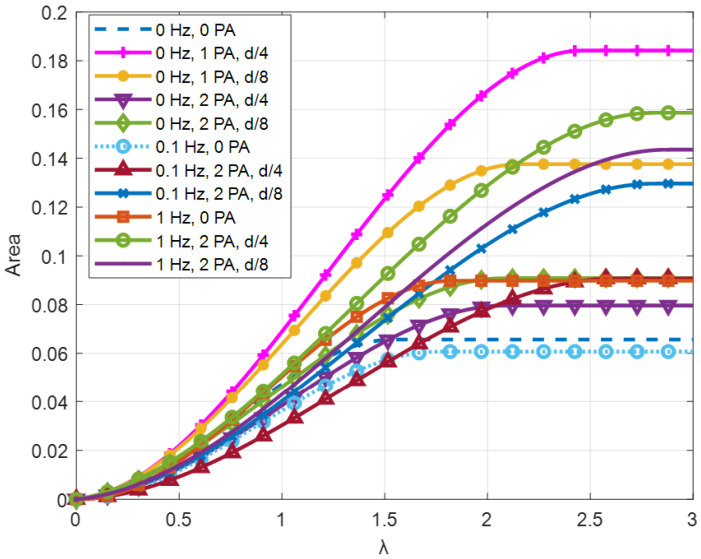
Comparison of area values between different configurations.

The integral function of the areas under the *C_P_–λ* curves for all tested configurations were plotted in Figure 17. Every curve has an inflection point in correspondence with the maximum value of the tip speed ratio; therefore, after that point, the value of the area remains stable. The final value of the area is proportional to the energy that can be harvested with a certain configuration.

**Figure 18:**
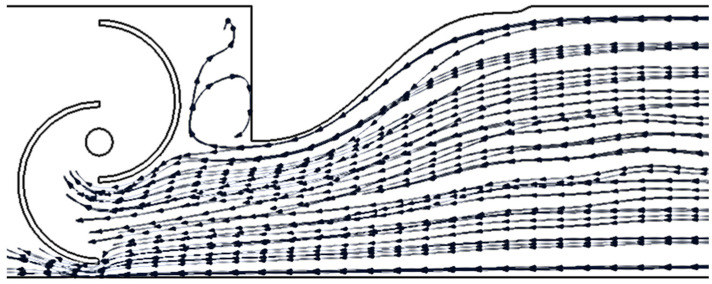
A streamlined representation of the physics with D/8 configuration.

**Figure 19:**
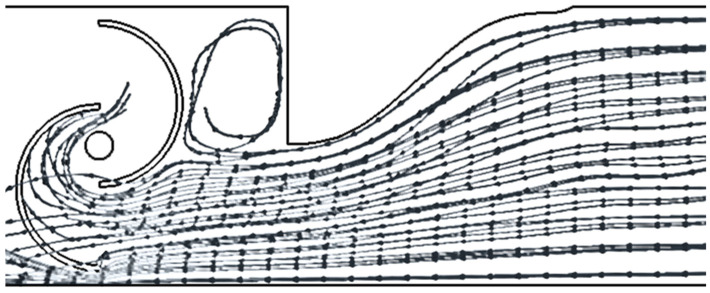
A streamlined representation of the physics with D/4 configuration.

The great advantage of the presence of the power augmenter is explained thanks to the streamlined example shown in the following pictures. Thanks to the power augmenter the streamlined areas are concentrated along the active part of the turbine. In addition, the PA generates a vortex zone (depressurized part) in the passive part of the turbine, which, if properly utilized, can function as a suction system that creates a positive effect in terms of rotation. However, if used incorrectly, it can generate disturbance. Therefore, it is crucial to act by considering the appropriate distance relative to this effect. A comparison between Figure 18 and Figure 19 shows that at the chamber’s rear, the PA can have different effects. The streamlined areas in the first case can interact directly with the turbine blade producing a great disturbance; in the case of *D*/4, in the chamber, there is space for vortex formation and thus for the creation of a low-pressure area.

**Figure 20:**
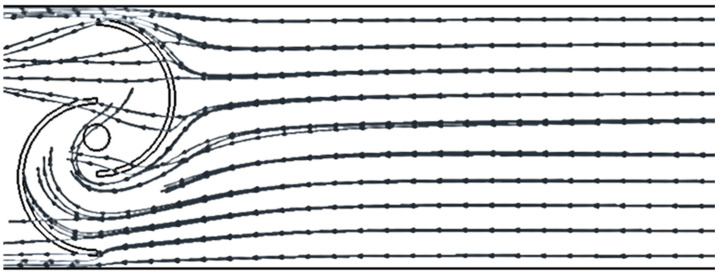
A streamlined representation of the physics without power augmenter.

The last picture (Figure 20) shows the case without a PA. The streamlined areas are equally distributed along the duct and they impact both blades of the turbine (active and passive side). This leads to a reduction in the efficiency of the turbine.

### 3.2. ML Results

The ML model based on decision trees, described in the previous section, underwent the phases of training, validation (using 10-fold cross-validation), and testing. The results are presented in Table 5 showing the values of *MSE*, *RMSE*, *R*^2^, and *MAE* obtained both during the validation and testing phases.

**Figure 21:**
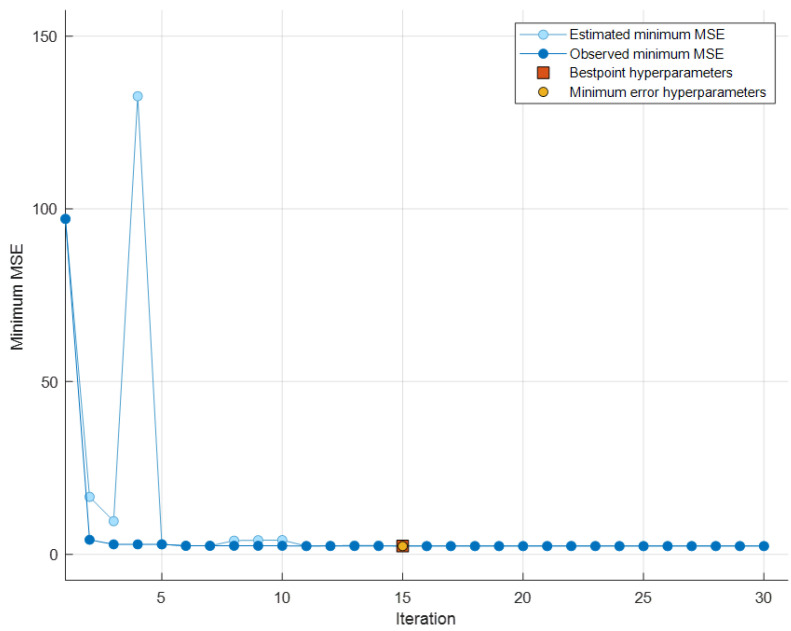
Minimum *MSE* values during the iterations and best point hyperparameters.

As previously mentioned, the metric to minimize is the *MSE*. From Figure 21, it can be seen that, during the 30 iterations conducted while varying the hyperparameters (during training and validation), the *MSE* was evaluated, reaching its minimum value at iteration number 15.

In this case, the minimum *MSE* coincides with the best combination of hyperparameters. Therefore, the model was constructed using this combination of hyperparameters, which are summarized in Table 6.

**Figure 22:**
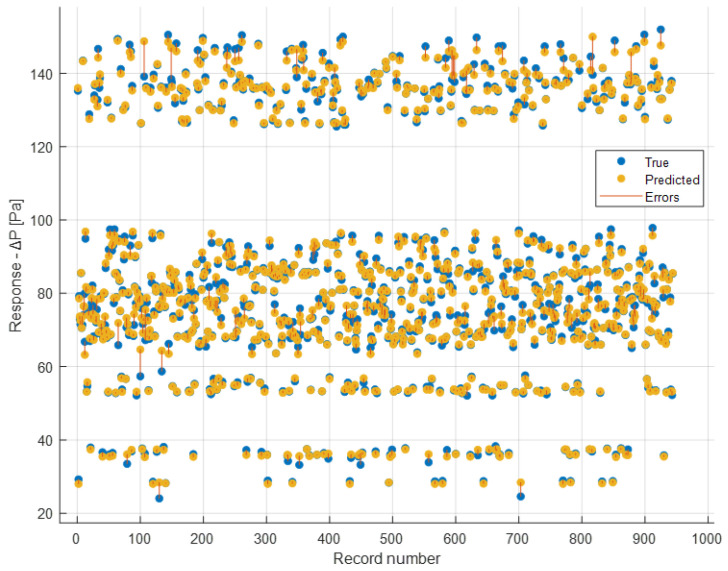
Comparison between true and predicted responses for each instance.

Figure 22 displays the plot correlating the 944 actual Δ*P* values for each instance (X-axis) with those predicted during the validation phase (Response).

They are depicted by blue dots (true) and yellow dots (predicted), respectively. Additionally, prediction errors are shown with red lines. Since cross-validation was performed, validation was conducted on all 944 instances.

**Figure 23:**
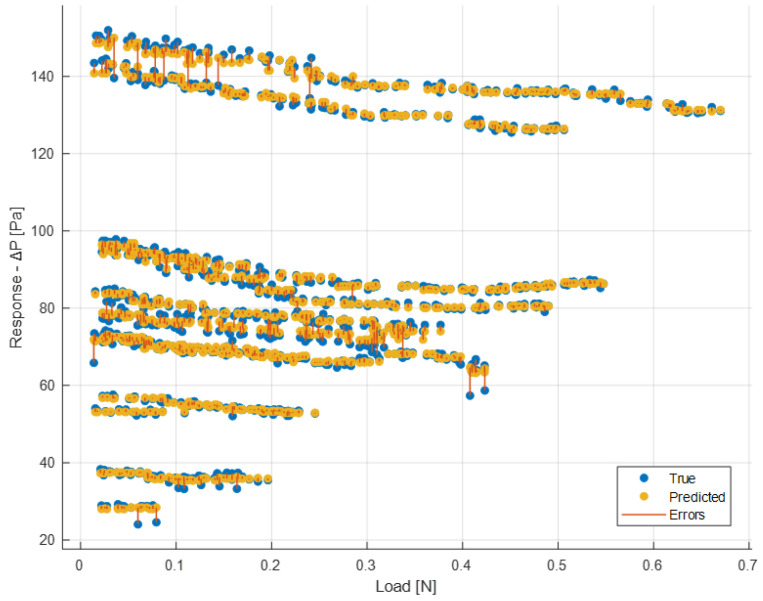
Comparison between true and predicted responses for each load value.

**Figure 24:**
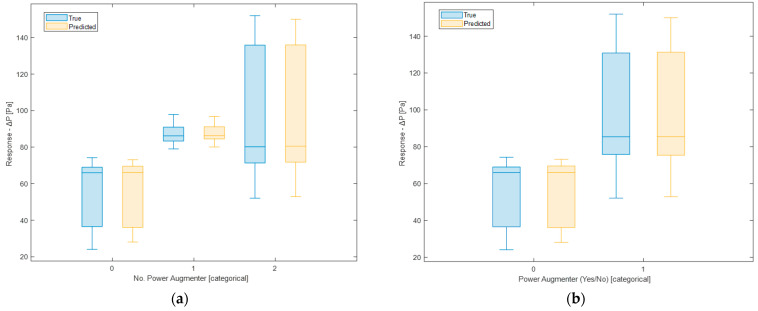

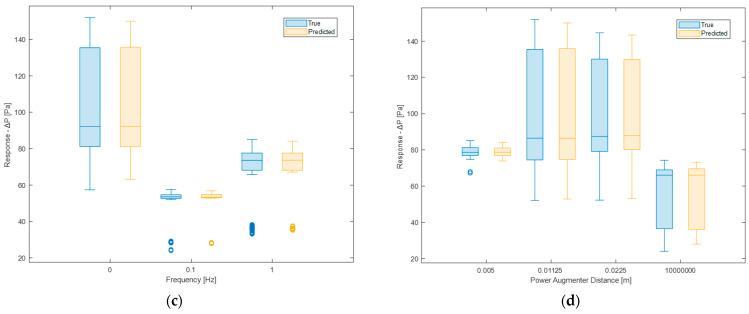
Comparison between true and predicted responses for each No. PA (**a**) and PA (yes/no) (**b**) categories and for each frequency (**c**) and PA distance (**d**) values.

It is also possible to have a graphical representation similar to the previous one but evaluated for each single feature. Figure 23 and Figure 24 show a comparison between the true and the predicted instances.

In the previous figures, therefore, the model’s responses in terms of Δ*P* are compared to the true ones. It is possible to observe a sufficient level of closeness, ensuring the goodness of the model as already indicated by the evaluation metrics shown earlier. In the ideal case (*R*^2^ = 1, perfect predictions), the box plot of the true responses and that of the predicted ones should be exactly the same. The values being compared are those of Δ*P*. Therefore, for example in Figure 22 the red line indicating the error depicts exactly the difference between the predicted values and the actual values. As regards the box plot, the central mark denotes the median, while the lower and upper edges of the box represent the 25th and 75th percentiles, respectively. Vertical lines extend from the boxes to the most extreme data points that are not considered outliers. Outliers are individually plotted using the o″ symbol.

**Figure 25:**
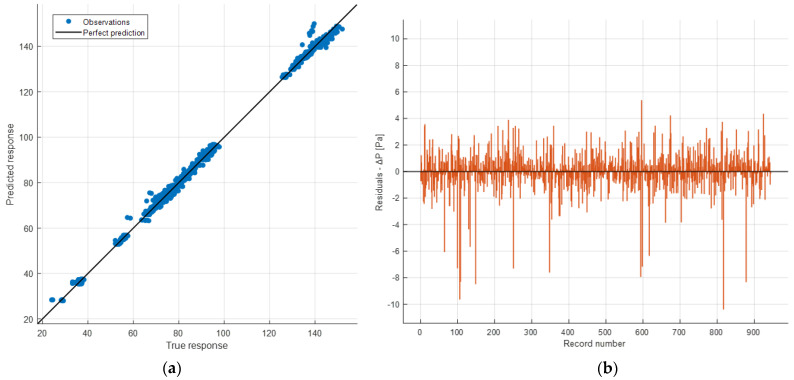
Prediction accuracy (*R*^2^ = 0.99767) (**a**) and residual plot (**b**) of the validation phase.

Figure 25a displays the correlation between the actual Δ*P* values (X-axis) and the predicted ones (Y-axis) in the validation phase. The plot is summarized by the *R*^2^ coefficient, which, as shown in Table 5, is sufficiently high.

In the ideal case, the predicted Δ*P* values coincide with the actual ones. In this scenario, all points lie on the line, resulting in an *R*^2^ coefficient equal to 1.

Figure 25b displays the residual plot for the 944 observations (the difference between the predicted and the true values of Δ*P*). It exhibits a certain symmetry around axis 0, except for some outliers in the negative part of the plot, and no pattern could be observed. This indicates good performance during the validation phase.

**Figure 26:**
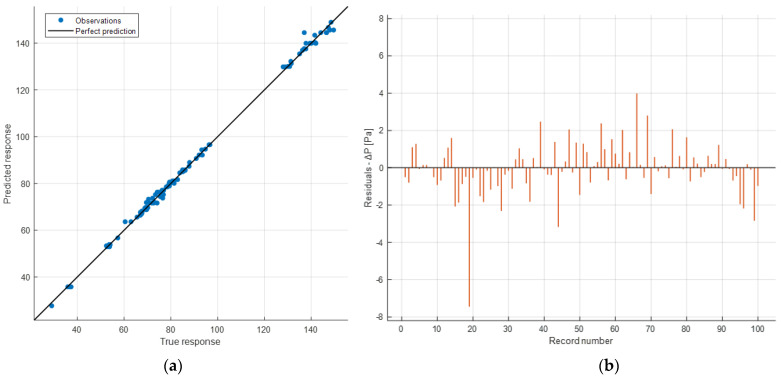
Prediction accuracy (*R*^2^ = 0.99801) (**a**) and residual plot (**b**) of the test phase.

Below, some graphs obtained as a result of the test phase are shown. Figure 26a displays the correlation plot between the predicted and the actual values of Δ*P*.

In the test phase, there are 100 instances, and from the distribution, it is noticeable how the observations approximate the line of perfect predictions better. This is also evident from the fact that the test *R*^2^ is closer to 1 compared to that of validation. This can be interpreted as a good performance of the model, as it has managed to achieve a prediction level comparable to that of validation. This means that the model has generalized well enough to predict new instances with the same level of accuracy. Therefore, hyperparameter optimization has, as expected, reduced the occurrence of overfitting.

Figure 26b displays the residual plot of the test data. Again, apart from an outlier value, the plot exhibits values distributed around zero.

Now, the graphs of partial dependencies of individual features on the response (Δ*P*) are shown.

The partial dependence plot illustrates the marginal effect that one or two features have on the prediction of a machine-learning model [50]. A partial dependence plot can show whether the relationship between the output and a feature is linear, monotonic, or more complex.

**Figure 27:**
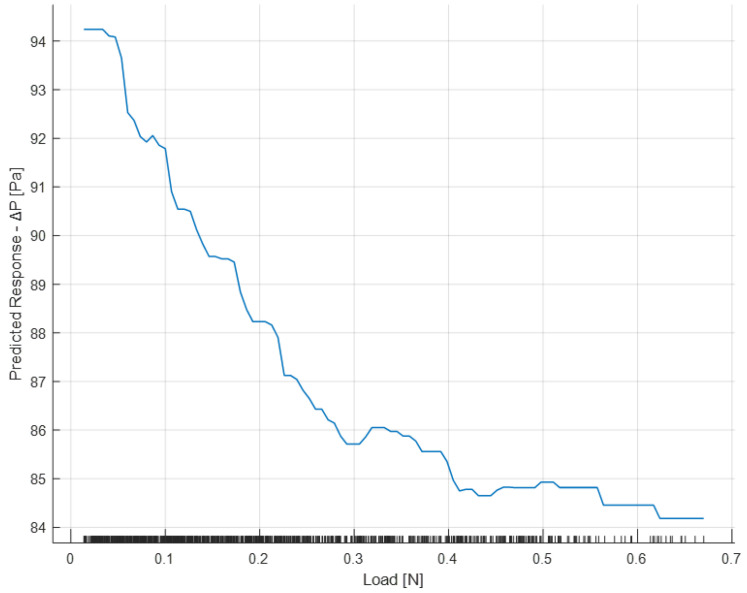
Partial dependence plot of the load.

In the partial dependence plot of the load (Figure 27), it can be observed that as the load applied to the turbine increases, the Δ*P* decreases in a quadratic manner. This can be justified by the fact that as the braking load increases, the turbine generates a progressively lower Δ*P*.

**Figure 28:**
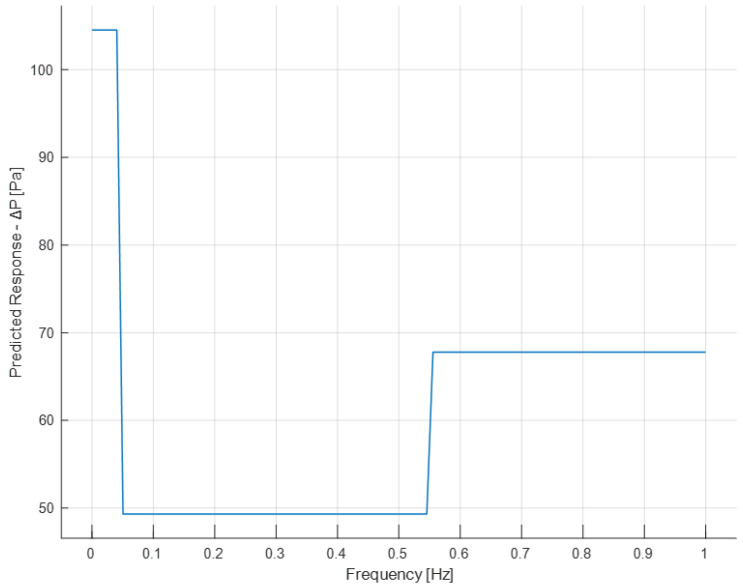
Partial dependence plot of the frequency.

The partial dependence plot of frequency (Figure 28) shows that for a frequency equal to zero (continuous flow), the Δ*P* has a high value, while when the flow becomes alternating, there is a rapid decrease in Δ*P*, which increases as it moves from lower to higher frequencies. However, the highest Δ*P* value during bi-directional flow remains significantly below the Δ*P* value at a continuous flow (0 Hz).

**Figure 29:**
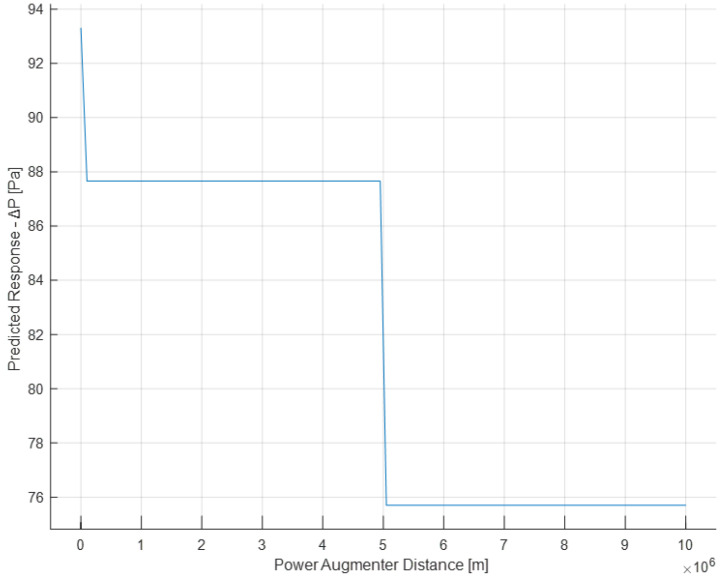
Partial dependence plot of the PA distance.

From the following graph (Figure 29), it is evident that increasing the PA distance and the turbine results in a significant decrease in Δ*P* from small to large distances (distance values of 10^6^ denote, in practice, the absence of PA).

In the case of categorical features, partial dependence is obtained by forcing all data instances to belong to the same category. Therefore, a value will be obtained for each class.

**Figure 30:**
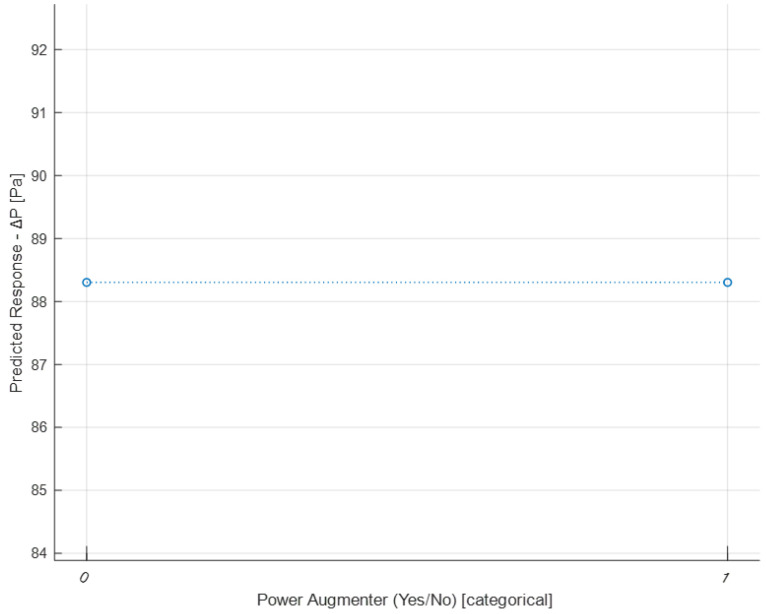
Partial dependence plot of the PA (yes/no).

Figure 30 displays the partial dependence plot concerning the presence/absence of PAs. As can be seen, neither of the two categories prevails over the other in terms of influence on Δ*P*. Consequently, this feature appears to be irrelevant in determining Δ*P*. This was partly predictable since, for example, in the previous graph (Figure 29), the PA distance = 10^7^ had already been appropriately chosen to represent the absence of a PA. Additionally, the feature No. PA encompasses both the absence and presence of PAs, providing additional information. However, it was decided to verify this hypothesis. Discarding this feature will not affect the obtained result. It is clarified that what has just been said does not imply that the presence or absence of PAs does not influence Δ*P* (as evidenced by Figure 29 and Figure 30), but only that it does not make sense to use this type of predictor in the model, as it is correlated with two other predictors.

**Figure 31:**
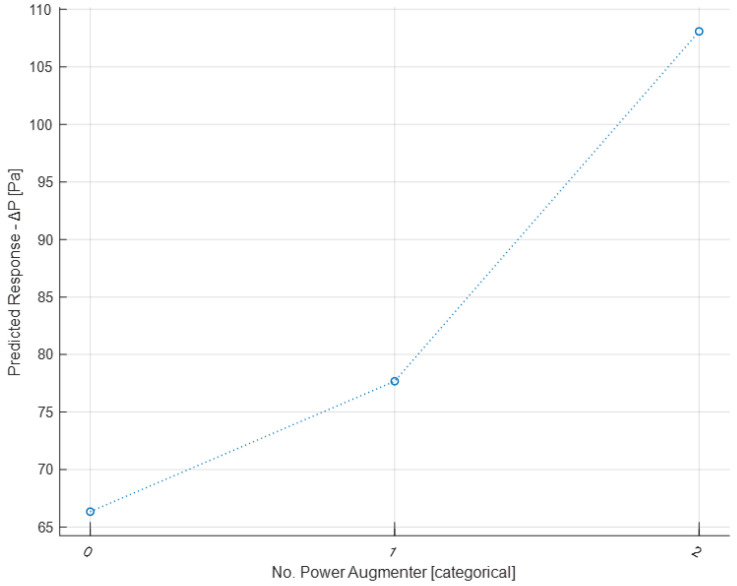
Partial dependence plot on the No. PAs.

From the partial dependence plot regarding the No. PAs present (Figure 31), it can be observed that the presence of even one PA has a positive impact on Δ*P*. With two PAs present, Δ*P* increases significantly compared to the previous case. It is therefore inferred that the presence of PAs, especially two PAs, leads to a higher Δ*P*.

**Figure 32:**
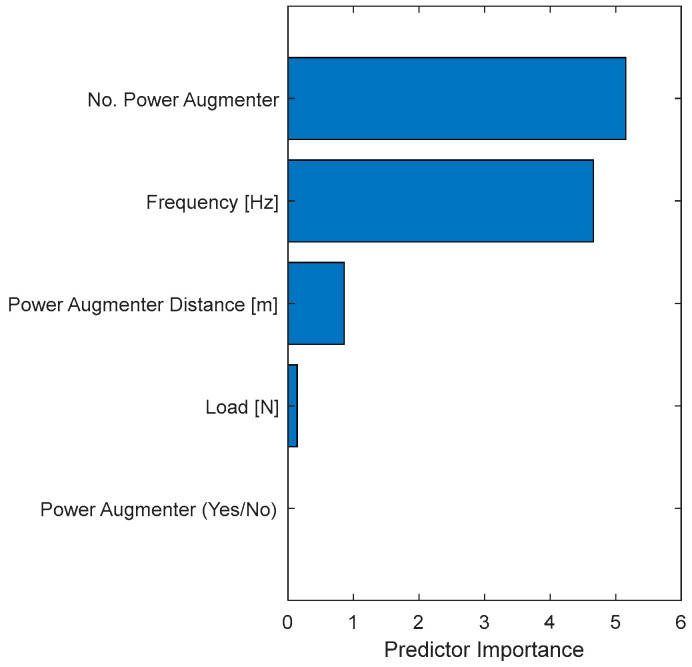
Predictor importance bar plot.

Furthermore, it is possible to extract the importance of the selected features (predictors) in determining the Δ*P* (as shown in Equation (18)). Figure 32 displays, in descending order, the predictors’ importance.

The most important features observed during model training are the number of PAs and frequency, which are of comparable importance. A lower weight is associated with the PA distance and the applied load. The feature regarding the presence/absence of PAs appears to have no weight. This is consistent with the previous consideration of Figure 30.

The greater importance of the “No. PA” feature is dictated by the operating mode of the PAs. In fact, the presence of the PAs ensures that the flow is appropriately directed toward the driving blade, creating a converging duct that increases the flow velocity. In particular, the unidirectional configuration benefits from the positive effect on Δ*P* of the presence of a single PA. In the bi-directional flow configuration, however, a PA placed downstream of the turbine creates a negative effect on Δ*P* as its vertical wall obstructs the flow. Nevertheless, a positive effect prevails over the negative one. This can also be observed by comparing the *C_P_–λ* curves at 0 Hz (Figure 11 with those at non-zero frequencies (Figure 10 and Figure 13), looking at the maximum *C_P_* values obtained (higher in the case of bi-directional flow).

As for frequency, it is natural that Δ*P* is strongly dependent on the flow frequency since it is the forcing wave of the system.

The influence of the PA distance is justified by the fact that it must assume precise values to have a globally positive effect on Δ*P*. Indeed, too large distances nullify the effect of directing the flow onto the driving blade, while distances that are too small create fluid dynamic conditions that worsen performance (as already shown in Figure 18, Figure 19 and Figure 20).

**Figure 33:**
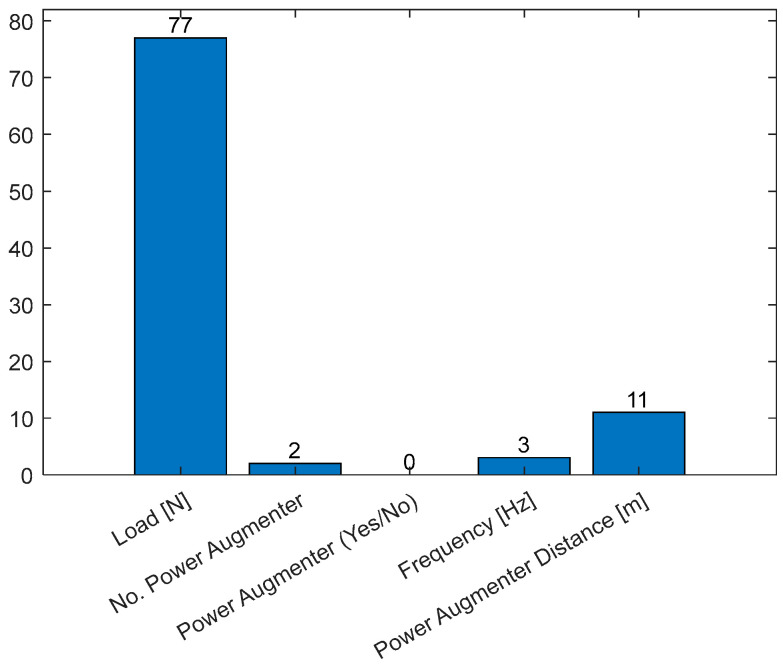
Count of the features used to split nodes.

Figure 33 shows the counts of the features used to generate the tree.

Figure 33 shows a complementary graph of Figure 32. The high counts of splits for the load [N] denote a great difficulty for the algorithm to minimize the cost function (MSE) with this feature, while with the other ones, the algorithm needs a very low split count to minimize the MSE. As expected, the binary predictor PA (yes/no) was never utilized to create a split within the tree. An inverse trend between the frequency of a feature’s use and its importance is observable (Figure 32. This confirms the general understanding that the most frequently used feature does not always have the greatest influence on determining the output (Δ*P*).

## 4. Conclusions

The main objective of the paper was to understand how a specially designed device (PA) can be employed to enhance the efficiency of a Savonius turbine while maintaining the same available power input. In this phase, comparative results among different solutions on the same turbine were presented to begin understanding the effects of these devices. The primary goal of the device is to increase the area of the *C_P_–λ* curve without needing to modify the geometry of the turbine. This could potentially allow for interventions on already installed Savonius turbines without needing to dismantle the rotor, but by acting upstream and downstream of the turbines. Another important result was leveraging a large number of tests (1044) to train a decision tree, providing a tool that can immediately offer responses regarding the *C_P_–λ* curves if one of the input parameters used by AI is varied. In particular, the experimental part of this study aimed to characterize turbine behavior by extracting *C_P_–λ* curves from experimental results. These curves represent the relationship between the power coefficient (*C_P_*) and the tip speed ratio (*λ*). The area under a *C_P_–λ* curve gives an estimate of the energy recoverable from wave motion. This area can be increased by either accelerating turbine rotation or improving the power coefficient.

For a flow frequency of 0 Hz, the results show that with a unidirectional flow, the greatest improvement occurs with 1-PA configurations, particularly as the proximity of the PA to the turbine increases. However, 2-PA configurations do not improve power due to downwind PA disturbance, though they do increase the tip speed ratio.

For a frequency of 0.1 Hz, adding 2 PAs significantly increases the power coefficient at a distance of *D*/8, making it the optimal configuration among those tested.

Similarly to the 0.1 Hz condition, when *f* = 1 Hz, the best performance is achieved with 2 PAs at *D*/8 from the turbine, though the improvements in power coefficient and tip speed ratio compared to the no-PA case are not as substantial.

Regarding the development of the predictive model, it was observed that among the features used, the greatest importance in determining Δ*P* is attributable to the No. PAs. Another feature of comparable importance is the fluid flow frequency. It was also noted that one of the selected features, the presence of PAs (yes/no), has no impact on determining the output as it is inherently correlated with two other features (PA distance and No. PAs). Therefore, it does not affect the model training and can be excluded. Obtaining a predictive model opens up the possibility of conducting further tests without the need to resort to the physical system, allowing for the evaluation of different scenarios. As future developments, training the model under additional conditions could be considered, always subject to obtaining experimental data, such as at different speeds. The PA can serve as a valuable asset in enhancing the efficiency of such turbines without necessitating a revolutionary overhaul, particularly for those already in operation. It can be integrated both upstream and downstream of the existing turbines. This research opens up avenues for various future developments. For instance, the experimental data could be utilized to calibrate a CFD model, enabling the optimization of the PA’s shape or the assessment of alternative profiles beyond the Bell–Metha design. Additionally, detailed studies, again employing CFD, can explore the optimal positioning of the devices and strategies for managing the vortices exiting these PAs. The study underscores the potential for enhancing these turbines through purpose-built, easily manufacturable devices.

## Figures and Tables

**Table 1:** Turbine characteristics.

Parameter	Symbol	Value
Diameter [mm]	*D*	90
Height [mm]	*h*	90
Overlap Ratio	*OR*	1/3
Aspect Ratio	*AR*	1
Spacing Ratio	*SR*	0
Axis diameter [mm]	*AD*	10
Axis length [mm]	*AL*	60
Weight [kg]	*W*	0.635

**Table 2:** Tested configuration.

Configuration Number	Number of PAs	PA Distance	Frequency [Hz]
1	0		0
2	0		0.1
3	0		1
4	1	*D*/4	0
5	1	*D*/8	0
6	2	*D*/4	0
7	2	*D*/4	0.1
8	2	*D*/4	1
9	2	*D*/8	0
10	2	*D*/8	0.1
11	2	*D*/8	1

**Table 3:** Setup variables with description and range of values.

Load [N]	No. PA	PA (Presence/Absence)	Fan Frequency [Hz]	Distance PA–Turbine [mm]
0.0138–0.6701 (continuous)	0, 1, 2 (categorical)	1, 0 (categorical)	0–1 (continuous)	0.0117–10^7^ (continuous)

**Table 4:** Datasets and number of instances.

Dataset	No. Instances
Total	1044
Training/Validation	944
Test	100

**Table 5:** Performance (validation and testing) of the trained ML model.

	*MSE*	*RMSE*	*R* ^2^	*MAE*
Validation	2.3773	1.5419	0.99767	1.0226
Test	1.9539	1.3978	0.99801	0.93791

**Table 6:** Best combination of the hyperparameters selected.

Hyperparameter	Value
Minimum Leaf Size	8
Maximum No. Split	943
Minimum Parent Size	16

## Data Availability

Data are available upon request.
